# Genetic variation in the tau protein phosphatase-2A pathway is not associated with Alzheimer's disease risk

**DOI:** 10.1186/1756-0500-4-327

**Published:** 2011-09-07

**Authors:** José L Vázquez-Higuera, Ignacio Mateo, Pascual Sánchez-Juan, Eloy Rodríguez-Rodríguez, Ana Pozueta, Miguel Calero, José L Dobato, Ana Frank-García, Fernando Valdivieso, José Berciano, Maria J Bullido, Onofre Combarros

**Affiliations:** 1 Neurology Service and CIBERNED, "Marqués de Valdecilla" University Hospital (University of Cantabria and IFIMAV), Santander, Spain; 2 Spongiform Encephalopathies Unit, National Microbiology Centre and CIBERNED, Carlos III Health Institute, Madrid, Spain; 3 Alzheimer Disease Research Unit, CIEN Foundation, Carlos III Health Institute, Alzheimer Center Reina Sofia Foundation, Madrid, Spain; 4 Neurology Service and CIBERNED, Hospital Universitario La Paz (U.A.M.), Madrid, Spain; 5 Molecular Biology Department and CIBERNED, Centro de Biología Molecular Severo Ochoa (C.S.I.C.-U.A.M.), Madrid, Spain

## Abstract

**Background:**

Tau abnormal hyperphosphorylation and the formation of neurofibrillary tangles in AD brain is the result of upregulation of tau kinases and downregulation of tau phosphatases.

**Methods:**

In a group of 729 Spanish late-onset Alzheimer's disease (AD) patients and 670 healthy controls, we examined variations into a set of candidate genes (PPP2CA, PPP2R2A, ANP32A, LCMT1, PPME1 and PIN1) in the tau protein phosphatase-2A (PP2A) pathway, to address hypotheses of genetic variation that might influence AD risk.

**Results:**

There were no differences in the genotypic, allelic or haplotypic distributions between cases and controls in the overall analysis or after stratification by age, gender or APOE ε4 allele.

**Conclusion:**

Our negative findings in the Spanish population argue against the hypothesis that genetic variation in the tau protein phosphatase-2A (PP2A) pathway is causally related to AD risk

## Background

**Figure 1:**
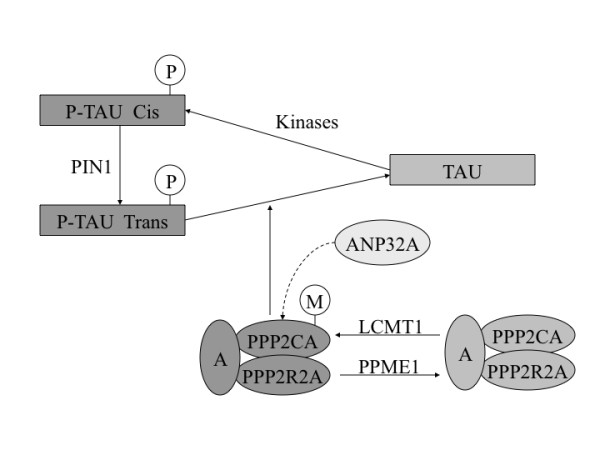
**Genes related with the tau protein phosphatase-2A (PP2A) pathway**. PP2A dephosphorylates tau and is composed of an structural subunit A, a catalytic subunit alpha (PPP2CA) and a regulatory subunit B alpha (PPP2R2A). The peptidyl-prolyl cis/trans isomerase PIN1 binds to tau and facilitates PP2A activity, whereas ANP32A inhibits PP2A activity. Methylation by the leucine carboxyl methyltransferase-1 (LCMT1) increases PP2A activity, and demethylation by protein phosphatase methylesterase-1 (PPME1) reduces PP2A activity.

One of the neuropathological hallmarks in Alzheimer's disease (AD) is neurofibrillary tangles (NFTs), which are composed of the microtubule-binding protein tau that is hyperphosphorylated [1]. Tau phosphorylation is catalysed by tau protein kinases and reversed by tau protein phosphatases [2]. It has been reported that the expression and activity of the major tau phosphatase in human brain, protein phosphatase-2A (PP2A), is decreased in the affected areas of AD brain [3,4], suggesting that a downregulation of tau phosphatases in AD brain might underlie the abnormal hyperphosphorylation of tau. The overall PP2A activity is determined by composition of the holoenzyme from the catalytic subunit alpha (PP2CA) and the regulatory subunit B alpha (PP2R2A), the level of PP2A inhibitors such as ANP32A (inhibitor-1 of protein phosphatase-2A), and changes in PP2A methylation regulated by the leucine carboxyl methytransferase-1 (LCMT1) and the protein phosphatase methylesterase-1 (PPME1); in addition, the peptidyl-prolyl cis/trans isomerase PIN1 induces conformational changes in tau that can facilitate tau dephosphorylation by PP2A (Figure 1). Consequently, PPP2CA, PPP2R2A, ANP32A, LCMT1, PPME1 and PIN1 are good candidate genes for the analysis of AD susceptibility. The largest genome wide association (GWA) study in AD [5] did not find significant results for all these PP2A-related genes. However, it cannot be discarded that these genes in the tau PP2A pathway are among the genes with significant nominal association but without reaching significance (p < 10^-5 ^or less) after adjustment for multiple testing in GWAs; in addition, it is also possible that some of the SNPs analyzed in our study were not present in the arrays used in GWAs. Therefore, we conducted a case-control association study involving genes in the tau PP2A pathway in relation to AD risk, in a Spanish cohort.

## Methods

The study included 729 AD patients (67% women; mean age at study 77.2 years; SD 8.0; range 61-103 years; mean age at onset 73.3 years; SD 7.8; range 60-100 years) who met NINCDS/ADRDA criteria for probable AD [6]. All AD cases were defined as sporadic because their family history did not mention any first-degree relative with dementia. AD patients were recruited from the Departments of Neurology of University Hospital "Marqués de Valdecilla" (Santander, Spain) and Hospital "La Paz" (Madrid, Spain), and from Alzheimer Center Reina Sofia Foundation (Madrid, Spain). The large majority of patients were living in the community and had been referred by their general practitioner; few had been admitted from hospital wards or nursing home facilities. Control subjects were 670 unrelated individuals (64% women; mean age 78.3 years; SD 9.4; range 60-104 years) randomly selected from nursing homes. These subjects had complete neurologic and medical examinations that showed that they were free of significant illness and had Mini Mental State Examination scores of 28 or more, which were verified by at least one subsequent annual following-up assessment. The controls arose from the same base population as the cases. The AD and control samples were Caucasians originating from a limited geographical area in northern Spain (Santander) and from the central area of Spain (Madrid).

Blood samples were taken after written informed consent had been obtained from the subjects or their representatives. The study was approved by the ethical committees of the University Hospital "Marqués de Valdecilla", Alzheimer Center Reina Sofia Foundation, and the Hospital "La Paz". Genotyping of PPP2CA (rs7705319 and rs4958177), PPP2R2A (rs2046225, rs7823021, rs17055172, rs12676426 and rs2046223), ANP32A (rs2924633, rs1551345, rs1551344, rs1551342, rs11632936, rs2958405 and rs1551343), LCMT1 (rs8062337, rs277898, rs277886 and rs277892), PPME1 (rs10898966, rs2848557, rs500608 and rs544356), and PIN1 (rs2233678, rs1077220, rs2010457 and rs2287838) polymorphisms was performed using the iPLEX Gold assay on the MassArray system (Sequenom Inc., San Diego, USA). We used data from the HapMap project http://www.hapmap.org to select htSNPs capturing 100% of PPP2CA genetic variability, 75% of PPP2R2A, 95% of ANP32A, 95% of LCMT1, 75% of PPME1, and 80% of *PIN1 *genetic variability in Caucasians. SNPs were chosen among those with minor allele frequencies ≥ 5% using Haploview v3.2 software http://www.broad.mit.edu/mpg/haploview with an r^2 ^threshold of 0.8. PPP2R2A rs12676426 and PPME1 rs500608 that were significantly deviated from Hardy-Weinberg equilibrium (HWE) were excluded from the analysis. In addition, we removed PIN1 rs1077220 with a non-homogeneous genotypic distribution between our two control populations (Santander and Madrid) and which were also different from the HapMap CEU distribution.

HWE was calculated for the htSNPs in the control population using Pearson's χ^2 ^statistics. We assessed pairwise linkage disequilibrium (LD) between the htSNPs by D' and r^2 ^statistics. Haplotype reconstruction and their frequencies in cases and controls were estimated by an expectation-maximization algorithm, method implemented in Haploview 3.32. Pearson's χ^2 ^statistics were performed to compare genotype, allele, and haplotype distribution of the patients and control for each htSNP. Genotypic and allelic distributions were assessed by logistic regression using SPSS software.

## Results

As shown in Table 1, the distribution of the minor allele frequencies of the PP2A-related genes did not differ significantly between AD and control groups. Haplotype distributions were not significantly different between cases and controls in the overall analysis or after stratification by APOE ε4 allele (data not shown). There were no major differences in allele, genotype or haplotype frequencies in our total sample associated to either age or gender subgroups. The data set was analyzed for epistatic interactions between the 6 PP2A-related genes and no significant effects were observed.

**Table 1:** Minor allele frequencies distribution of tau phosphatases genes in AD patients and controls

Gene	SNP	MAF, AD/C	*P*-value	Gene	SNP	MAF, AD/C	*P*-value
PPP2CA	rs7705319	0.20/0.20	0.99	LCMT1	rs8062337	0.32/0.30	0.34
	rs4958177	0.11/0.10	0.46		rs277898	0.37/0.38	0.35
					rs277886	0.34/0.32	0.16
PPP2R2A	rs2046225	0.38/0.37	0.49		rs277892	0.28/0.27	0.40
	rs7823021	0.28/0.29	0.88				
	rs17055172	0.15/0.17	0.31	PPME1	rs10898966	0.11/0.09	0.24
	rs2046223	0.38/0.39	0.82		rs2848557	0.34/0.34	0.84
					rs544356	0.20/0.20	0.89
ANP32A	rs2924633	0.40/0.40	0.67				
	rs1551345	0.09/0.08	0.48	PIN1	rs2233678	0.11/0.12	0.47
	rs1551344	0.07/0.06	0.31		rs2010457	0.33/0.32	0.83
	rs1551342	0.11/0.12	0.57		rs2287838	0.48/0.49	0.84
	rs11632936	0.27/0.27	0.97				
	rs2958405	0.31/0.34	0.07				
	rs1551343	0.13/0.11	0.20				

*P *-values not corrected for multiple comparisons.

## Discussion

Genes harbouring markers with only modest evidence of association (nominally significant but not reaching the genome-wide significance threshold) can be identified if they belong to the same biological pathway or mechanism; therefore, pathway-based approaches, which jointly consider multiple variants in interacting or related genes, might complement the most-significant SNPs/genes approach for interpreting genome-wide association (GWA) data on complex diseases [7,8]. In fact, genetic variation in the immune system and in lipid metabolism pathways is a cause of AD susceptibility [9,10]. Although genetic markers of the genes examined in this study (PPP2CA, PPP2R2A, ANP32A, LCMT1, PPME1 and PIN1) were not found associated to AD in the largest GWA study [5], our main hypothesis was that common variation in genes directly related to tau dephosphorylation in the PP2A pathway might underlie individual differences in susceptibility to AD. To our knowledge, this study is the first of its type to be conducted in this pathway. There is a selective and significant neuron-specific reduction in PP2A catalytic subunit and PP2A regulatory B subunit mRNAs in AD hippocampus [3,4], and this reduced neuronal PP2A immunoreactivity closely correlates with NFT load [4], suggesting that PP2A dysfunction contributes to AD tau pathology. In addition, cDNA microarray techniques have revealed downregulated expression of PPP2CA gene in AD brain [11,12]. Conversely, in AD brain has been observed a significant increase in the neocortical levels of PP2A inhibitors [13], which co-localize with abnormally hyperphosphorylated tau. These data suggest the possible involvement of ANP32A (inhibitor-1 of PP2A) in AD neurofibrillary pathology through the inhibition of PP2A activity. LCMT1 promotes PP2A activity by methylating its catalytic subunit, and PPME1 catalyses the removal of the methyl group, thus reversing the activity of LCMT1. Methylated PP2A catalytic subunit levels are reduced in AD frontal/temporal cortex [14], and LCMT1 levels are also selectively decreased in AD-affected regions and in tangle-bearing neurons [15]. An interaction between tau and PIN1 is thought to facilitate the dephosphorylation of hyperphosphorylated tau by PP2A, promoting microtubule stability [16]. In AD brain, PIN1 expression in the hippocampus and parietal cortex is low [17] and inversely correlated with neurofibrillary degeneration [18].

In an Italian study [19], carriers of PIN1 (-842, rs2233678) C allele had an increased risk of AD, lower age of onset, and reduced PIN1 levels in peripheral mononuclear cells, but subsequent studies [20-23] did not replicate these findings. We failed to detect the association of PIN1 (-842, rs2233678) with AD. We also failed to observe any allele, genotype or haplotype association of PPP2CA, PPP2R2A, ANP32A, LCMT1 and PPME1 genes with AD. Because we studied htSNPs capturing 80% of PIN1 and 75% of PPP2R2A and PPME1 genetic variability, it might be argued that we have missed a hypothetical disease locus, which would have been detected by analysis of extended haplotypes; however, the complete linkage disequilibrium across the PIN1, PPP2R2A and PPME1 regions in our study argues against this possibility. Our negative results with all these PP2A pathway-related genes are probably not due to insufficient statistical power, because our sample size had enough power (94%) to detect and odds ratio of 1.5 at disease allele frequencies of 0.10.

## Conclusion

Despite supporting evidence for the biological role of tau phosphatases in AD exists, our negative findings in the Spanish population argue against the hypothesis that genetic variation in the tau protein phosphatase-2A (PP2A) pathway is causally related to AD risk.

## Competing interests

The authors declare that they have no competing interests.

## Authors' contributions

JLVH and ERR performed the genetic studies and reviewed critically the manuscript. PSJ performed the statistical analyses and reviewed critically the manuscript. IM, AP, MC, JLD, AF, FV, JB and MJB reviewed critically the manuscript. OC drafted the manuscript and contributed to its final version. All authors read and approved the final manuscript.
